# Ιnclusion Complexes of Magnesium Phthalocyanine with Cyclodextrins as Potential Photosensitizing Agents

**DOI:** 10.3390/bioengineering10020244

**Published:** 2023-02-13

**Authors:** Eleni Kavetsou, Charalampos Tsoukalas-Koulas, Annita Katopodi, Alexandros Kalospyros, Eleni Alexandratou, Anastasia Detsi

**Affiliations:** 1 Laboratory of Organic Chemistry, School of Chemical Engineering, National Technical University of Athens, 15780 Athens, Greece; 2 Laboratory of Biomedical Optics and Applied Biophysics, School of Electrical and Computer Engineering, National Technical University of Athens, Zografou Campus, 15780 Athens, Greece

**Keywords:** phthalocyanine, cyclodextrins, inclusion complexes, kinetic modeling, photodynamic therapy

## Abstract

In this work, the preparation of inclusion complexes, (ICs) using magnesium phthalocyanine (MgPc) and various cyclodextrins (β-CD, γ-CD, HP-β-CD, Me-β-CD), using the kneading method is presented. Dynamic light scattering (DLS) indicated that the particles in dispersion possessed mean size values between 564 to 748 nm. The structural characterization of the ICs by infrared spectroscopy (FT-IR) and nuclear magnetic resonance (NMR) spectroscopy provides evidence of the formation of the ICs. The release study of the MgPc from the different complexes was conducted at pH 7.4 and 37 °C, and indicated that a rapid release (“burst effect”) of ~70% of the phthalocyanine occurred in the first 20 min. The kinetic model that best describes the release profile is the Korsmeyer–Peppas. The photodynamic therapy studies against the squamous carcinoma A431 cell line indicated a potent photosensitizing activity of MgPc (33% cell viability after irradiation for 3 min with 18 mW/cm^2^), while the ICs also presented significant activity. Among the different ICs, the γ-CD-MgPc IC exhibited the highest photokilling capacity under the same conditions (cell viability 26%). Finally, intracellular localization studies indicated the enhanced cellular uptake of MgPc after incubation of the cells with the γ-CD-MgPc complex for 4 h compared to MgPc in its free form.

## 1. Introduction

Phthalocyanines (PCs) are large, aromatic, organic molecules that consist of a large internal porphyrazine ring, which connects four isoindole units linked together by nitrogen atoms. PCs display a wide range of applications due to their chemical, electronic, structural, and optical properties [1,2]. There is also the possibility for incorporation of a metal atom (Mg, Zn etc.) in the center of their ring to form molecules known as metallophthalocyanines, which results in improved properties. Phthalocyanines and metallophthalocyanines have been initially used as pigments and coloring substances as well as chemical sensors, semiconductors, etc. [3]. PCs are now being studied as promising photosensitizing agents for photodynamic therapy (PDT) of both cancer and non-cancer related diseases [4,5,6,7].

Nowadays, PDT is being investigated thoroughly as an alternative to conventional treatment of both cancer and non-cancer related diseases. During PDT, a non-toxic dye (the photosensitizer) is administered and then it is exposed to a light source of a specific wavelength depending on the absorption properties of the molecule used [8]. The interaction of light with the photosensitizer leads to the death of the target cells by oxidative stress, which is induced after the stimulation of the photosensitizer and subsequent production of reactive oxygen species (ROS). PCs are considered as 2nd generation photosensitizers along with porphyrins and chlorins [3,4]. Their structure and properties render them excellent candidates for PDT applications, while their synthesis is relatively simple. PCs can also be structurally modified in order to modify their properties, such as their hydrophobicity. As molecules, they tend to be stable and are able to produce significant amounts of ROS. In regard to their photophysical properties, the absorbance maximum is noted to be at approximately 675 nm which is within the therapeutic window and, more importantly, at the red region of the visible spectrum where the penetration of the light in human tissues is reported to be the maximum. However, PCs are not hydrophilic molecules and display extremely low solubility in aqueous media, tending to form aggregations due to *π*-π* stacking and hydrophobic interactions [3,4].

Recent studies focus on the encapsulation of phthalocyanines in different carriers and the evaluation of their properties regarding their respective future application in order to overcome the aforementioned disadvantages [9,10,11,12]. More specifically, polymeric nanoparticles have been used in order to co-load curcumin along with phthalocyanines as a novel drug delivery system used in PDT [9], while other studies have developed multifunctional hybrid nanoparticles consisting of PLGA and hyaluronic acid in order to encapsulate phthalocyanines and investigated their tumor targeting effect as well as their photodynamic activity [10]. Liposomes have also been studied as promising carriers for phthalocyanines: in the work of Nkanga et al., cyclodextrins and liposomes were combined in order to successfully encapsulate an isoniazid-hydrazone-phthalocyanine conjugate [11]. In this context, a very promising candidate that has attracted attention the last few years and was investigated in this study as well, are cyclodextrins (CDs) and their ability to form inclusion complexes (ICs) [13,14].

**Figure 1:**
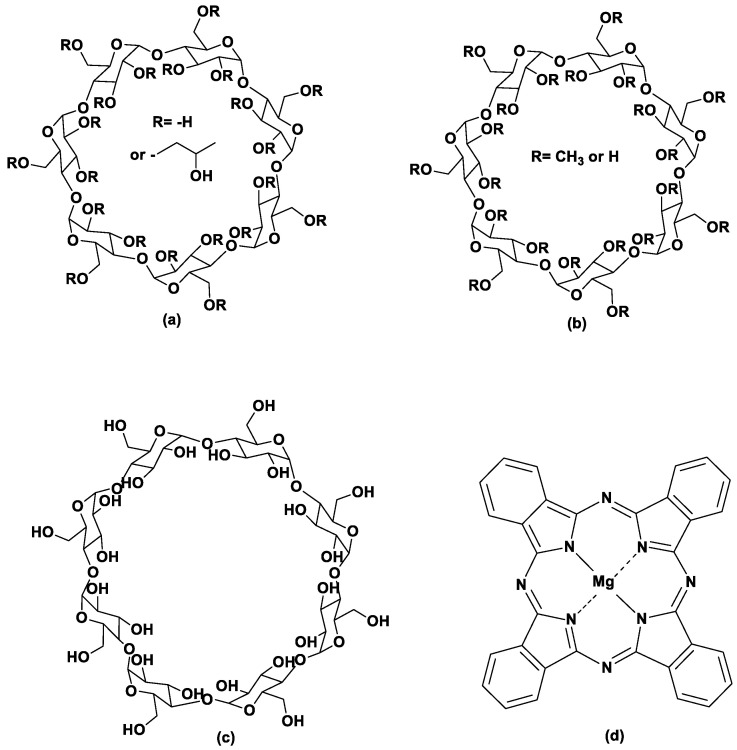
Structures of (**a**) 2-Hydroxypropyl-β-cyclodextrin, (**b**) methyl-β-cyclodextrin, (**c**) γ-cyclodextrin, and (**d**) magnesium phthalocyanine.

Cyclodextrins (CDs) are a family of cyclic oligosaccharides, derived from starch and they consist of repeating α-D-glucopyranose units. The number of α-D-glucopyranose units is six, seven, and eight for α-CD, β-CD, and γ-CD, respectively [15,16,17]. The structure of the CDs resembles a truncated cone with a hydrophilic outer surface and a hydrophobic inner cavity. At the narrow ring of the cone, the primary -OH groups of the 6-CH_2_OH group of the glucopyranose ring are located, whereas at the ring with the wider diameter, the secondary -OH groups are found. Due to the aforementioned structure, CDs are water soluble and can form water-soluble inclusion complexes with guest molecules which can be entrapped into the hydrophobic cavity. In an aqueous environment, hydrophobic molecules, such as phthalocyanines, or a part of them, can replace the water molecules inside the cavity leading to a new thermodynamic equilibrium [16,18]. Chemically modified cyclodextrins, such as hydroxy-propyl-β-CD (HP-β-CD), methylated β-CD (Me-β-CD) (Figure 1), and the sulfobutyl ether derivative of β-CD (SBE-β-CD) have been also used as host molecules as they present higher aqueous solubility than the natural β-CD [19].

The purpose of the present work was initially to study the inclusion of magnesium phthalocyanine (MgPc) (Figure 1) in natural (β-CD, γ-CD) and chemically modified cyclodextrins (HP-β-CD, Me-β-CD) in order to increase the solubility of MgPc and potentially enhance their photodynamic activity. The ICs were characterized and the release profile and kinetic modeling of MgPc release from the inclusion complexes were determined. The resulting inclusion complexes were then studied in terms of their optical properties and their ability to produce reactive oxygen species (ROS). PDT activity of free MgPc and its ICs was evaluated in vitro against an A431 skin cancer cell line, and the intracellular localization of the most potent photosensitizing nanosystem was also studied.

## 2. Materials and Methods

### 2.1. Materials

β-Cyclodextrin (β-CD), HP-β-Cyclodextrin (HP-β-CD), and Me-β-Cyclodextrin (Me-β-CD) of >99% purity were purchased from Honeywell Fluka (Charlotte, NC, USA), ACROS (Waltham, MA, USA) and Glentham (Corsham, UK), while magnesium phthalocyanine (MgPc) was purchased from Alfa Aesar (Haverhill, MA, USA), 5-(and-6)-chloromethyl-2′-7′dichlorodi-hydrofluorescein diacetate acetyl ester (CM-H_2_DCFDA) was purchased from Molecular Probes (Eugene, OR, USA). Ethanol of analytical reagent grade was purchased from Merck Millipore (Burlington, MA, USA) and dimethyl sulfoxide (DMSO) of ACS grade was purchased from Chem-Lab (Zedelgem, Belgium). All the dispersions and solutions were prepared using deionized water. The aforementioned materials were used without further purification. Dulbecco’s Modified Eagle Medium (DMEM), high glucose, and Dulbecco’s phosphate buffered saline (DPBS), without CaCl_2_ and MgCl_2_, pH 7.4, were purchased from Gibco (Waltham, MA, USA). Fetal bovine serum (FBS), antibiotic–antimitotic, and gentamicin were also purchased from Gibco. Trypsine (EDTA), Dimethyl sulfoxide (DMSO), and 3-(4,5-dimethylthiazol-2-yl)-2,5-diphenyl-2H-tetrazolium bromide (MTT) were obtained from Sigma-Aldrich (St. Louis, MO, USA). The human epidermal carcinoma cell line A431 was obtained from the American Type Culture Collection (ATCC) (Manassas, VA, USA).

### 2.2. Preparation of Magnesium Phthalocyanine Inclusion Complexes (ICs) with Various Cyclodextrins

Magnesium phthalocyanine was incorporated in various cyclodextrins using the kneading method [19,20]. MgPc and the respective cyclodextrin (β-CD, γ-CD, HP-β-CD, Me-β-CD) were added to the mortar in molar ratio 1:1. To create a homogeneous paste, a solution of water:ethanol in ratio 3:2 was added dropwise. The paste was ground for at least 45 min. The final blue solid powder was dried using a high vacuum pump and stored under refrigeration, for further analysis and characterization. The same procedure (kneading method) was applied under solvent-free conditions for MgPc and HP-β-CD, while the corresponding physical mixture of the components was also prepared for the better assessment of the ICs’ formation (prepared samples were used for comparison purposes of the FT-IR and/or NMR analysis).

### 2.3. Evaluation of the Stoichiometry of the ICs Using Job’s Plot

To determine the stoichiometry of the inclusion complex, the Job’s plot was indicatively conducted for β-CD/MgPc mixture. Phosphate buffer solutions (pH 7.4) with known concentrations of both compounds (MgPc and β-CD) were prepared. The total sum of the two masses was kept constant while their molar ratio varied from 0 to 1 (Equation (1)). The absorbance of each sample was determined using UV-Vis. With these measurements a (Abs(MgPc) − Abs(MgPc + β − CD)) versus molar ratio graph was constructed. The stoichiometry was evaluated from identifying the maximum or minimum point in the graph.

(1) $$\mathrm{Molar}\;\mathrm{ratio}\;\left(r\right)=\frac{\left[\mathrm{MgPc}\right]}{\left[\mathrm{MgPc}\right]+\left[\beta -\mathrm{CD}\right]}$$

### 2.4. Characterization of the CD—MgPc ICs

#### 2.4.1. Process Yield

The process yield (PY) is used to determine the suitability of a process chosen for a specific protocol and is calculated by dividing the final mass of the dried inclusion complexes by the total amount of CD and MgPc initially used. For each IC that was produced, the PΥ was calculated from the following equation (Equation (2)).

(2) $$\%\mathrm{PY}=100\times \frac{\mathrm{mass}\;\mathrm{of}\;\mathrm{the}\;\mathrm{prepared}\;\mathrm{inclusion}\;\mathrm{complex}\;\left(\mathrm{mg}\right)}{\mathrm{initial}\;\mathrm{mass}\;\mathrm{of}\;\beta -\mathrm{CD}\;+\;\mathrm{initial}\;\mathrm{mass}\;\mathrm{of}\;\mathrm{MgPc}\;\mathrm{to}\;\mathrm{be}\;\mathrm{encapsulated}\;\left(\mathrm{mg}\right)}$$

#### 2.4.2. Inclusion Efficiency of the CD-Phthalocyanines ICs

The inclusion efficiency (IE) describes the percentage of the encapsulated MgPc in the CD—MgPc ICs, relative to the total starting amount of MgPc used ((Equation (3)).

(3) $$\;\%\mathrm{IE}\;=100\times \frac{\mathrm{mass}\;\mathrm{of}\;\mathrm{the}\;\mathrm{encapsulated}\;\mathrm{MgPc}\;\left(\mathrm{mg}\right)}{\mathrm{initial}\;\mathrm{mass}\;\mathrm{of}\;\mathrm{MgPc}\;\mathrm{to}\;\mathrm{be}\;\mathrm{encapsulated}\;\left(\mathrm{mg}\right)}$$

The inclusion efficiency of the MgPc in the CD-MgPc ICs was calculated using UV-Vis by determining the quantity of the encapsulated MgPc. All of the UV-Vis measurements were performed on a V-770 UV-Vis Jasco spectrophotometer. For each dried inclusion complex, 10 mL of dimethyl sulfoxide (DMSO) was added into 10 mg of IC and stirred for at least 24 h at room temperature. The samples were then filtered, diluted appropriately, and their absorbance was measured in the range of 500–800 nm. All of the measurements were performed in triplicate.

#### 2.4.3. Dynamic Light Scattering (DLS)

In order to determine the size, polydispersity index, and the *ζ*-potential of the CD-MgPc ICs by the DLS method, measurements were performed on the Zetasizer Nano ZS Malvern. The samples were prepared by dispersing 1 mg of the CD-MgPc ICs in 20 mL of double deionized water. The final solutions were then stirred and vortexed directly before the measurement. For the measurements, cuvettes of type U (DTS1070) were used. For each sample, the measurements were performed at 25 ± 1 °C and in triplicate. The results are reported as mean ± standard deviation. All of the measurements were conducted for pH 7.4.

#### 2.4.4. Fourier Transform Infrared Spectroscopy (FT-IR Spectroscopy)

FT-IR measurements were performed in order to study the formation of the ICs as well as the interaction between the MgPc and the cyclodextrins. A JASCO FT/IR-4200 spectrometer (Japan Spectroscopic Company, Tokyo, Japan) was used. All the measurements for MgPc, CDs, the final dried ICs, the physical mixture, and the solvent-free kneading product were conducted in the form of KBr pellets, in the scanning range of 650–4000 cm^−1^.

#### 2.4.5. Nuclear Magnetic Resonance Spectroscopy (NMR Spectroscopy)

^1^H-NMR spectra of CDs, the final dried CD-MgPc ICs, and the physical mixture of HP-β-CD and MgPc were obtained using the Varian 600 MHz spectrometer (Varian, Palo Alto, CA, USA), located at the Institute of Chemical Biology, National Hellenic Research Foundation). The samples were dissolved in deuterated DMSO (DMSO-*d_6_*). The chemical shifts were expressed in parts per million (ppm) while the coupling constants (*J*) in hertz (Hz).

### 2.5. In Vitro Release Studies of the MgPc from the CD-MgPc ICs

The release profile of the MgPc was evaluated under specified conditions. For each IC, 5mg of the dried final product was added to different glass vials, each one corresponding to a specific time of incubation. In each vial, 2 mL of phosphate buffer (pH 7.4) was added, and the samples were kept in an incubator under 37 °C. At predetermined time intervals, each vial was removed, filtered, and diluted with DMSO, in order to determine the MgPc concentration. All of the measurements were done in duplicate.

#### Kinetic Modeling of the MgPc Release from the CD-MgPc ICs

The kinetic models that are widely applied to describe the release of bio-active substances from drug delivery systems are the zero-order model, first-order model, Higuchi model, and Korsmeyer-Peppas model [21,22].

From the Korsmeyer-Peppas equation, the diffusion exponent (n) can be calculated. After the calculation of n, the mechanism of the release can be described.

(4) $$F=\frac{M_{t}}{M}=k_{m}t^{n}$$

which can also be written as:

(5) $$\log\left(\frac{M_{t}}{M}\right)=\log\left(k_{m}\right)+nlog\left(t\right)$$

Based on the aforementioned equations (Equations (4) and (5)), the slope of the log(Mt/M) versus log(t) graphs is equal to the diffusion exponent n [21,22].

### 2.6. Optical Properties of Inclusion Complexes of MgPc with Natural and Modified CDs

#### 2.6.1. UV-Vis Absorption

The absorption spectra of MgPc in DMSO and CD-MgPc ICs in PBS (1% *v*/*v* DMSO) were recorded at the concentration of 2 μΜ using a Perkin-Elmer Lambda 35 UV/VIS spectrometer. The samples were freshly prepared just before measurements. All measurements were carried out at room temperature.

#### 2.6.2. ROS Production

CM-H_2_DCFDA is a chloromethyl derivative of 2′,7′-dichlorodihydrofluorescein diacetate (H_2_DCFDA). This substance works as fluorescent tracer and can be detected after being hydrolyzed because it turns into a fluorescent substance when it reacts with free radicals. The hydrolysis of CM-H_2_DCFDA was carried out using sodium hydroxide (NaOH).

Subsequently, the hydrolyzed substance is added in a PBS buffer solution (1% *v*/*v* DMSO) containing MgPc either in its free form or as ICs at the concentration of 5 μM. The solution was irradiated at 661 nm. Irradiation was performed using a 661 nm with a power density output of 14 mW/cm^2^. During irradiation, samples were constantly stirred using a magnetic stirrer. For the evaluation of the ROS production, the fluorescence spectrum of CM-H_2_DCFDA was recorded every 2 min for 10 min using a Perkin-Elmer LS45 luminescence spectrometer.

### 2.7. Cell Culture Conditions

The cells were cultivated in 75 cm^2^ culture flasks (Corning, Corning, NY, USA) in Dulbecco’s Modified Eagle Medium (DMEM), high glucose, supplemented with 10% heat inactivated fetal bovine serum (FBS), 1% antibiotic–antimitotic, 0.5% streptomycin-penicillin, and gentamicin. Cells were kept at 37 °C in a 5% CO_2_ humidified incubator, trypsinized, and re-seeded into fresh medium every 3–5 days.

### 2.8. Cell Viability Assessment, MTT Assay

Cell viability was evaluated by the MTT colorimetric assay, which measures the capacity of mitochondrial dehydrogenase to reduce MTT to purple formazan crystals. The MTT test assesses the number of surviving cells.

Cells were seeded in 96-well plates (6000 cells/well) and grown overnight at 37 °C in a 5% CO_2_ incubator. Exponentially growing cells were treated accordingly (dark toxicity, light toxicity, photodynamic treatment). Twenty-four hours after their treatment, the medium was removed and the MTT solution (0.65 mg/mL) was added to each well. The cells were kept in the incubator for 3 h to allow the metabolism of MTT and then the medium was removed and 200 μL of dimethyl sulfoxide (DMSO) was added, resulting in the solubilization of the formazan crystals. The absorbance was recorded at 570 nm (peak of formazan absorption spectrum) using an Epoch 2 microplate reader (Bio Tek Instruments, Winooski, VT, USA). The results were expressed as % cell viability = (mean optical density (OD) of treated cells/mean OD of untreated cells) × 100. All measurements were carried out in triplicate and all data were expressed as means ± standard deviation.

#### 2.8.1. Dark Toxicity Studies

After seeding of the cells in the 96-well plates and incubating for 24 h, the cells were treated with different concentrations (0.05 μM, 0.5 μM, 1 μM, 3 μM, and 5 μM) of MgPc in its free form or in the ICs for 24 h. Finally, cellular survival was measured with the MTT assay.

#### 2.8.2. Light Toxicity Studies

Twenty-four hours after seeding the cells in 96-well plates, the culture medium was removed from the wells and PBS was added (40 μL) so as to slightly cover the cells’ monolayer. The cells were then irradiated at 661 nm with power density at cellular level of 18 mW/cm^2^. Exposure times were 60 s, 120 s, and 180 s resulting in fluence rates of 1.08, 2.16, and 3.24 J/cm^2^, respectively. Following irradiation, fresh medium was added, and the cells were maintained in the humidified incubator for 24 h. Finally, cellular viability was assessed via MTT method as described previously.

### 2.9. Photodynamic Treatment

After their seeding for 24 h, cells were incubated with 0.5 μM of freshly prepared solutions of MgPc and its cyclodextrin complexes in enriched medium for 4 h. In continuation, the medium containing the photosensitizers was removed, 40 μL of PBS were added in each well, and the cells were irradiated with fluence rates of 1.08, 2.16, and 3.24 J/cm^2^ as indicated for the light toxicity studies. Following irradiation, fresh medium was added, and the cells were incubated for another 24 h. Finally, cell viability was measured with the MTT assay.

### 2.10. Irradiation Device

Irradiation was performed using a 661 nm diode laser system coupled to an optical fiber and a light diffuser (GCSLS-10-1500m, China Daheng Group, Beijing, China) in order to provide a uniform circular illumination spot. At each experimental condition, three wells were irradiated, and the laser spot was centered to them. Before and after cellular irradiation, laser power was measured at the cellular level using a power meter. Irradiance variability in different points of the irradiated area was less than 2%.

### 2.11. Intracellular Localization

A431 cells were seeded in glass coverslips (10 × 10^4^ cells) and incubated overnight in 2.5 mL of complete medium (DMEM). Cells were subsequently treated with MgPc or its cyclodextrin complexes (0.5 μM final concentration) for 4 h. Cells were observed under an epifluorescent upright microscope, Olympus BX-50 (Olympus Optical Co., GmbH, Hamburg, Germany), using a 40× objective lens (UPlanFl, N.A. = 0.75, Olympus, Hamburg, Germany) coupled to a CCD camera (XC-30, Olympus, Hamburg, Germany). Image acquisition was performed using the analySIS getIT software version 5.1 (Olympus Soft Imaging Solutions GmbH, Hamburg, Germany) [23].

## 3. Results

### 3.1. Evaluation of the Stoichiometry of the ICs Using Job’s Plot

**Figure 2:**
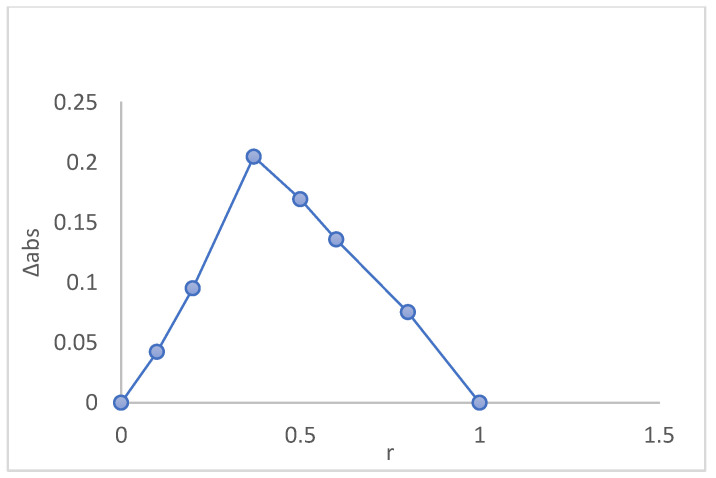
Job’s plot figure to determine the stoichiometry of the β-CD-MgPc IC.

The Job’s plot diagram is presented in Figure 2. Τhe maximum for the molar ratio (r) is determined to be 0.33. This value indicates that the stoichiometry of this IC is 1:2 MgPc/β-CD (Figure S1). This result is also compliant with experiments conducted in similar studies [24] for β-CD ICs with chemically modified zinc phthalocyanines.

### 3.2. Characterization of the CD-MgPc ICs

#### 3.2.1. Process Yield and Inclusion Efficiency

The process yield values as well as the inclusion efficiency are presented in Table 1. For the determination of %IE, the calibration curve for MgPc in DMSO was used. The absorption was measured at 673.4 nm. The %PY and the %IE for each IC are shown in Table 1.

Based on the Table 1, β-CD displays the highest %PY, while HP-β-CD displays the highest inclusion efficiency followed by β-CD. Me-β-CD and γ-CD display lower encapsulation efficiency values.

#### 3.2.2. Size, Polydispersity Index (PDI), and ζ-Potential

For the ICs that were produced in this study, the size, the PDI, and the *ζ*-potential are shown in Table 2.

Using different CDs, the size and the stability of the system vary. The hydrodynamic diameter of the prepared ICs ranges from 564.5 ± 52.6 (for the β-CD-MgPc complex) to 748.7 ± 52.0 nm (for the γ-CD-MgPc complex). The PDI ranges from 0.522 to 0.566 in all cases and indicates moderately uniform distribution of the particles’ sizes. It is well known that CDs have the tendency to aggregate in aqueous solutions at room temperature merely because of the lack of sufficient charge on their surface. Usually, the aggregation does not take place in a uniform manner, thus affecting the PDI of the ICs. In regard to the *ζ*-potential, it is lower (absolute value) in the case of HP-β-CD-MgPc and γ-CD-MgPc (−17.7 ± 0.5 mV and −14.9 ± 4.0 mV, respectively) and higher in the case of β-CD-MgPc and Me-β-CD-MgPc (−29.8 ± 1.18 mV and −23.0 ± 1.6 mV, respectively).

These values indicate moderate to high stability of the particles in an aqueous dispersion [25,26]. More specifically, the ICs consisting of HP-β-CD and γ-CD, display moderate stability while the ICs with β-CD and Me-β-CD display higher stability.

#### 3.2.3. Fourier Transform Infrared Spectroscopy (FT-IR Spectroscopy)

FT-IR spectroscopy is widely used to investigate the interactions between the host molecule and the guest molecule [27,28]. In the FT-IR spectrum of pure MgPc, the most characteristic absorption bands that are observed are at 1525, 1483, 1333, 1057, 888, and 728 cm^−1^. The band at 1525 cm^−1^ is attributed to the C=C-C stretching of the aromatic ring, whereas the band at 1483 cm^−1^ is owed to the C-C stretching vibration of the isoindole structure. The bands at 1333 and 1057 cm^−1^ are owed to the C-C stretching and the C-N stretching vibrations of the pyrrole ring, respectively. At 888 cm^−1^ the absorbance can be attributed to the Mg-N stretching vibration, whereas the band at 727 cm^−1^ is owed to out of plane deformation of the C-H bond [29]. In Table 3, the characteristic absorption bands of the pure CDs as well as the peaks for the different ICs, the prepared physical mixture, and the solvent free kneading product are reported.

For all of the spectra of the prepared CD-MgPc ICs, a similarity with the spectrum of the pure CD used in each case is observed. In all of the ICs, there is a shift of the characteristic bands of the pure CDs, especially for the stretching vibration of the O-H group which presents the largest shift. Furthermore, there is a significant shift of the bands that were attributed to the antisymmetric stretch of the C-H bond of the CH_2_ group as well as to the bending vibration of the O-H group, which is an indication of the interaction between the different CDs and MgPc.

In addition, the characteristic absorption band of pure MgPc at 1525 cm^−1^ is shifted in the spectra of the ICs (β-CD-MgPc IC: 1521 cm^−1^, HP-β-CD-MgPc IC: 1528 cm^−1^, Me-β-CD-MgPc IC: 1521 cm^−1^), which also indicates an interaction between the two components. Finally, the characteristic band of pure MgPc at 728 cm^−1^ is still noticeable in the spectra for the ICs, which indicates that only part of the phthalocyanine is located in the interior part of the CDs. This result has also been noted in other studies [11,30], which concluded that only part of the phthalocyanine is inserted in the hydrophobic cavity of cyclodextrins.

The FT-IR spectra of the physical mixture of HP-β-CD and MgPc and of the corresponding solvent-free kneading product present significant similarities. It is noteworthy that all the characteristic absorption bands of MgPc are present in both spectra, whereas in the spectra of the ICs only a few of them are evident, which is indicative of the successful formation of the ICs when the appropriate preparation method is applied.

#### 3.2.4. Nuclear Magnetic Resonance Spectroscopy (NMR Spectroscopy)

**Figure 3:**
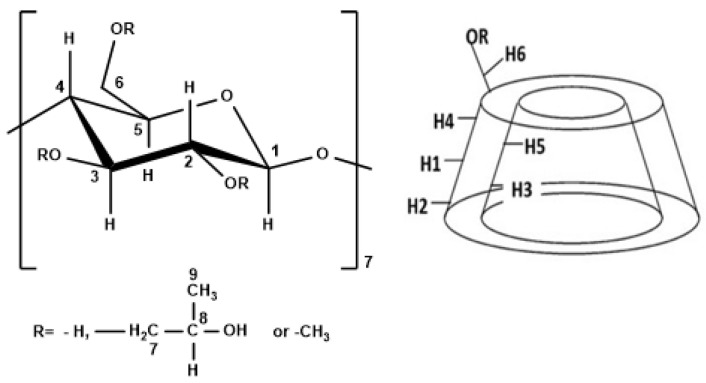
Left: numbering of β-glucopyranose monomer for the different CDs, right: structure of the CDs.

NMR spectroscopy is an extremely useful technique for studying the structure of organic compounds, especially in the case of ICs since it provides essential information on the structure of supramolecular host–guest complexes [31]. The NMR analysis for the ICs of CDs with MgPc and of the HP-β-CD and MgPc physical mixture was performed in DMSO-*d_6_* at 600 MHz. The structure of the β-CD monomer and its 3D depiction can be seen in Figure 3.

In the ^1^H-NMR spectra, the changes of the chemical shifts of the protons inside and outside of the cavity of the CDs can be identified, which can be informative regarding the inclusion mode and the affinity between the different CDs and MgPc.

The change of the chemical shifts for β-CD and the β-CD-MgPc ICs are presented in Table 4. A notable change of the chemical shift was identified for H-5, which is located inside the cavity of β-CD. Similar changes of the chemical shifts are observed for protons H-1, H-2, H-3, and H-6, from which H-3 and H-6 are located within the cavity while H-1 and H-2 are located outside the cavity (Figure 3). More specifically, all of the aforementioned protons displayed a downfield shift after the formation of the ICs, which is an indication of the proximity of β-CD to an electronegative atom, such as nitrogen in the structure of MgPc. Moreover, the observed shielding of H-5, H-3, and H-6, indicates that a part of the MgPc molecule interacts with the protons in the hydrophobic region of the host-molecule while another part of the MgPc molecule is located outside of the cavity, forming non-inclusion complexes or aggregates. This is in accordance with the FT-IR analysis results.

From Table 5, significant changes in chemical shifts are observed for H-4 and H-1 of Me-β-CD, both located on the outer surface of the cavity. It is also noteworthy that no significant differences in the chemical shifts are observed for the H-3 and H-5 protons that are located inside the Me-β-CD’s hydrophobic cavity. Moreover, the downfield shifts are an indication of the proximity of Me-β-CD to an electronegative atom, such as nitrogen in the structure of MgPc [32]. These data indicate that MgPc is retained at the exterior surface of Me-β-CD, forming non-inclusion complexes or aggregations, and interacting with the exterior oxygen atoms of Me-β-CD.

Based on the data of Table 6, significant changes in chemical shifts were observed for the H-2, H-4, and H-7 of HP-β-CD. These upfield shifts indicate that the MgPc molecule interacts with the protons that are located outside the cavity, as well as with the H-7 of the methylene group which is also located outside the cavity. As far as the protons that are located inside the cavity are concerned, a significant downfield shift is noted for the H-3 (Δδ = 0.024 ppm), while for H-5 the ∆*δ* is significantly smaller (Δδ = 0.008 ppm), indicative of the partial inclusion of the MgPc inside the cavity and the orientation of MgPc molecules towards the largest rim of the HP-β-CD. The downfield shift is possibly attributed to the proximity of HP-β-CD with an electronegative atom, such as nitrogen in the structure of MgPc [11,33].

However, an upfield shift is observed for protons H-3, H-5, and H-6 in the spectra of the physical mixture of HP-β-CD and MgPc (Table 7), along with lower Δδ compared to the HP-β-CD-MgPc IC. Moreover, in the ^1^H NMR spectrum of the physical mixture, a better signal splitting of the protons’ peaks of MgPc is clearly observed, whereas in the inclusion complex spectrum, a broadening of the peaks occurred (Figure S2). Thus, these observations revealed the successful formation of the HP-β-CD-MgPc IC when using the kneading method [33].

### 3.3. In Vitro Release Studies of the MgPc from the CD-MgPc ICs

**Figure 4:**
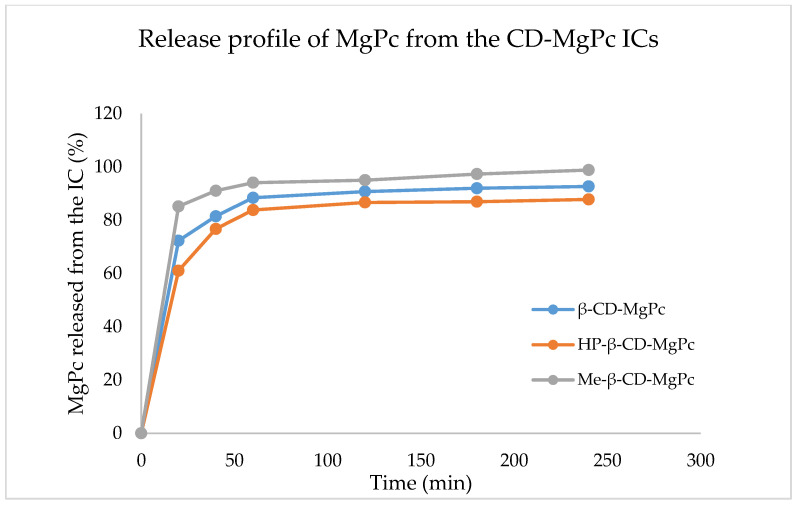
Release profile of MgPc from the different CD-MgPc ICs.

The determination of the release profile of a drug delivery system is of great significance since it provides vital information concerning its use for specific applications. The release profile of MgPc from the different ICs is shown in Figure 4.

As it is observed in Figure 4, for all the ICs, the release profiles display similar behavior. More specifically, all of the CD-MgPc ICs, display a rapid release (“burst effect”) of MgPc in the first 20 min. In this timeframe, 72%, 76%, and 85% MgPc is released for β-CD-MgPc, HP-β-CD-MgPc, and Me-βCD-MgPc, respectively. This rapid release is attributed to the diffusion of MgPc molecules located on the outer surface of the CDs due to weak interactions. After the burst effect there is a decrease in the rate of release (“lag effect”) and after 1h, there is a “plateau” indicating the sustained release of MgPc.

The IC that displays the fastest release of MgPc is the one with Me-β-CD, followed by β-CD, and lastly HP-β-CD. Especially in the case of the Me-β-CD-MgPc ICs, the fastest release profile is also in accordance with the ^1^H-NMR results which indicated that MgPc is mostly located on the surface of the Me-β-CD.

#### Kinetic Modeling of the MgPc Release from the CD-MgPc ICs

In Table 8, the equations for each model used in order to fit the data derived from the in vitro release analysis are presented. The fitting (R^2^) can be compared for each model in order to determine which one describes the release more efficiently. The most effective fitting of the data is noted when the Korsmeyer–Peppas model is used for all the different ICs.

**Figure 5:**
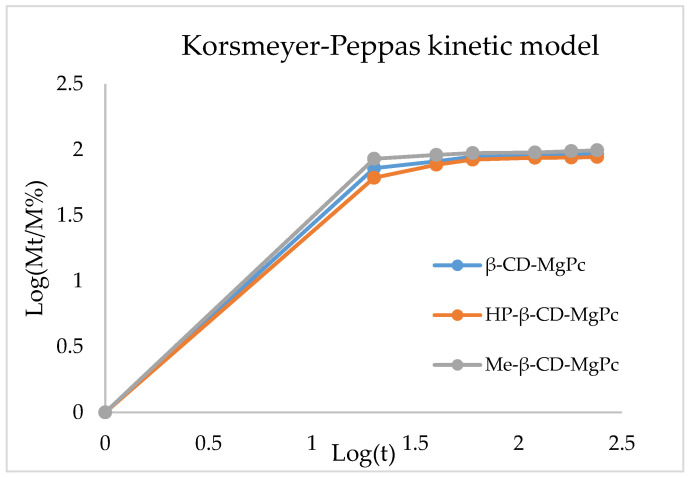
Graphical representation of the Korsmeyer-Peppas kinetic model of MgPc in vitro release.

The diffusion exponent n, as it is derived from the graphs for the different ICs, are presented in Table 9. The values of n indicate, that for all the ICs, the release mechanism is non-Fickian anomalous transport (Figure 5) [21,22].

### 3.4. Optical Properties of CD-MgPc ICs

#### 3.4.1. Absorption Spectroscopy

**Figure 6:**
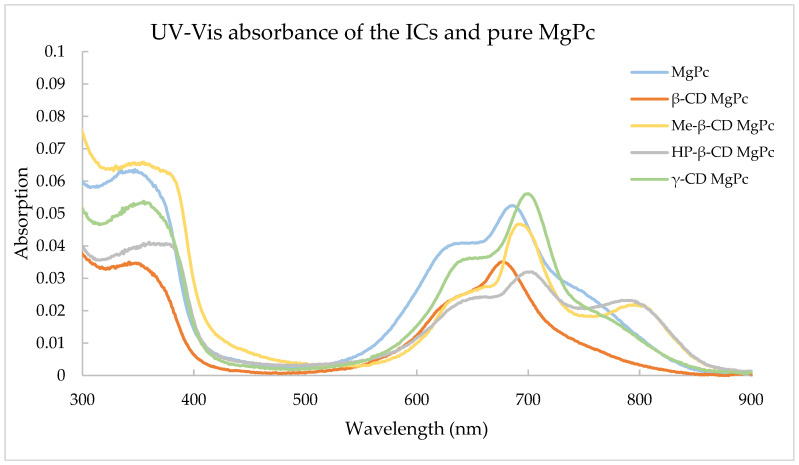
Absorbance of the ICs and pure MgPc.

Figure 6 displays the absorption spectra of pure MgPc and the corresponding ICs.

The dominant peak of MgPc is observed at approximately 670 nm, while this peak seems to have shifted to longer wavelengths for all ICs except for β-CD MgPc. This is due to the interaction of MgPc and the different CDs in order to form the supramolecular structure of the ICs. The dominant peak of phthalocyanines was also less visible after the complexation, which has been noted in other studies as well [34]. It is also worth mentioning that Me-β-CD MgPc and HP-β-CD MgPc also exhibited a new peak at a longer wavelength, which was not visible elsewhere in any of the other CD-MgPc ICs.

#### 3.4.2. ROS Production Evaluation

**Figure 7:**
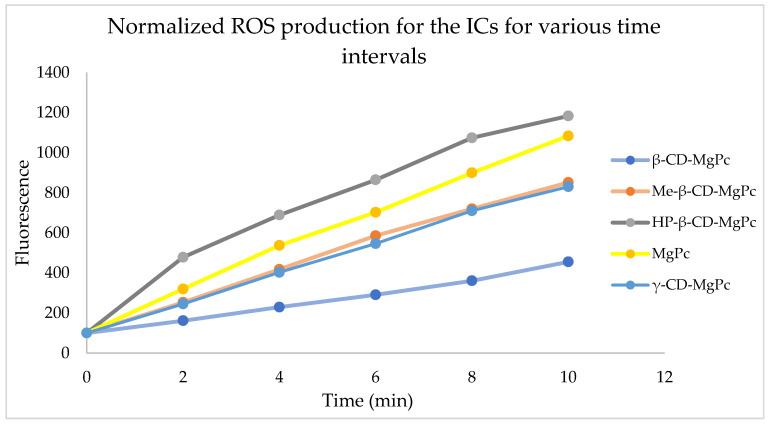
ROS production ability of the prepared CD-MgPc ICs at the concentration of 5 μM.

For studying the production of ROS from the ICs and the pure MgPc, the samples underwent laser exposure for specific timeframes and then fluorescent spectrophotometry was used at 490 nm, which is the excitation wavelength for the fluorescent tracer. In Figure 7, ROS production ability of MgPc and the different ICs is displayed for different time intervals. Data are presented as percentage of fluorescein intensity prior to irradiation.

Based on the above diagram, all the substances tested are able to produce ROS. More specifically, the largest ROS production, even higher than the one from pure MgPc, occurs from the IC with HP-β-CD. ICs with Me-β-CD and γ-CD present similar ROS production ability, while β-CD appear to be less active than the others. Based on that, the ICs consisting of modified CDs (Me-β and HP-β-CD) produce ROS faster in aqueous solutions when compared to the ICs prepared with the unmodified, natural β-CD and γ-CD. The increased ROS production capacity of the MgPc in the ICs with HP-β-CD could be attributed to the degradation of the host molecule, leading to the release of free MgPc in the solution which in turn causes higher ROS production after laser treatment [34].

### 3.5. Photodynamic Treatment

**Figure 8:**
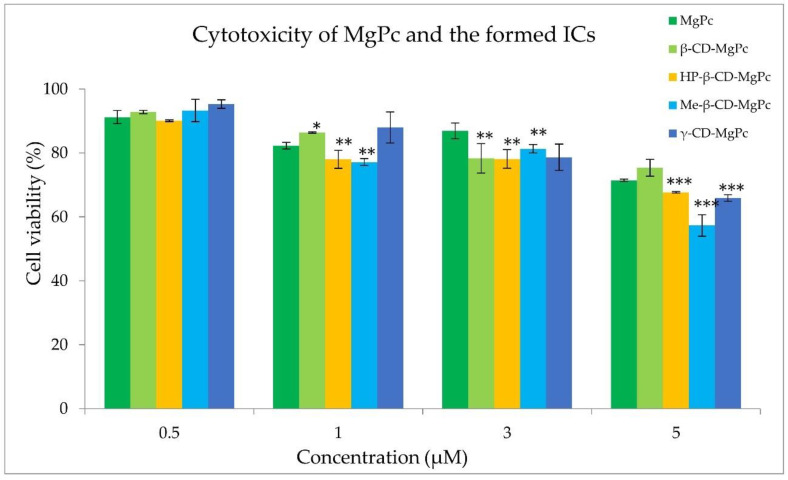
Effect of different concentrations of MgPc in free form or encapsulated in the different complexes on the A431 cell viability. * *p* < 0.05 versus control; ** *p* < 0.005 versus control; *** *p* < 0.0001 versus control.

In order to proceed with the photodynamic treatment studies, dark and light toxicity experiments were performed. The appropriate concentration of the photosensitizer should not exhibit cytotoxicity without irradiation, while the applied light energy dose in the absence of photosensitizer should not present cytotoxicity as well. The cytotoxicity results of different concentrations of MgPc against an A431 squamous carcinoma cell line, both in its free form and in complexation with the different cyclodextrins, are presented in Figure 8.

**Figure 9:**
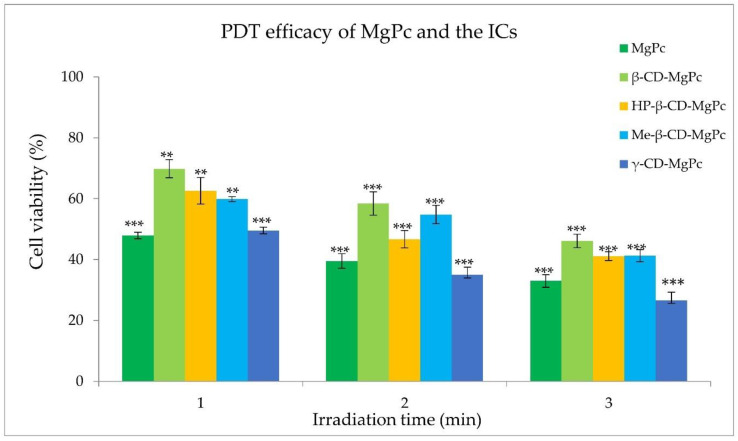
Effect of irradiation of MgPc and its cyclodextrin complexes (0.5 μM) with 18 mW/cm^2^ for 1 min, 2 min, and 3 min on the cell viability of A431 cell line. ** *p* < 0.005 versus control; *** *p* < 0.0001 versus control.

The examined concentrations of MgPc between 1 μM and 5 μM exhibited significant cytotoxicity, leading to their exclusion from further studies. However, cell treatment with a concentration of 0.5 μM of MgPc for 24 h did not affect the cell viability. In addition, light irradiation at 661 nm with power density of 18 mW/cm^2^ and fluence rates of 1.08, 2.16, and 3.24 J/cm^2^ was also found to be non-cytotoxic. Consequently, the photodynamic effect of MgPc and its cyclodextrin complexes against A431 cells was evaluated at the concentration of 0.5 μM, and with a power output density of 18 mW/cm^2^ for various irradiation times. The results obtained on cell survival are presented in Figure 9.

MgPc exhibited potent photosensitizing activity at the concentration of 0.5 μM under all irradiation conditions, reducing the cell viability to approximately 33% after 3 min of irradiation with 18 mW/cm^2^. All the examined CD-MgPc ICs presented significant PDT efficacy as well. The γ-CD-MgPc complex was the most promising photosensitizing nanosystem, presenting higher PDT activity than free MgPc, reducing the cell viability to 26% after 3 min of irradiation. This could be possibly attributed to the enhanced cellular uptake of MgPc after its encapsulation in the γ-CD cavity, improving its aqueous solubility and thus reducing its agglomeration [35]. On the other hand, complexation with the less water soluble β-CD [36], lead to an IC which presented the lowest phototoxicity under all the examined irradiation conditions (46% viability after 3 min of irradiation). This observation is in accordance with the ROS production studies in which β-CD-MgPc IC presented significantly lower ROS production ability than free MgPc and all the examined ICs. Overall, complexation of different phthalocyanines with cyclodextrins can be considered as an effective strategy for PDT efficiency improvement [37,38].

### 3.6. Intracellular Localization Studies

**Figure 10:**
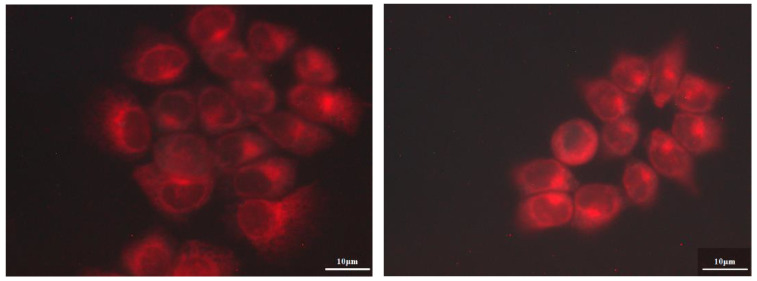
Fluorescence microscopic images of A431 cancer cells that were incubated for 4 h with 0.5 μM of MgPc (**left**) and γ-CD-MgPc IC (**right**).

Intracellular localization was studied using fluorescence microscopy. Indicative images of A431 cells incubated with 0.5 μM of MgPc and γ-CD-MgPc IC for 4 h are shown in Figure 10.

Any of the substances used did not affect cell structure and no nuclear localization was observed at 4 h of incubation. Fluorescence images presented higher fluorescence values in the case of cells incubated with γ-CD-MgPc compared to pure MgPc, indicating augmented cellular uptake of MgPc after its complexation with γ-CD. These findings are in accordance and can also explain the photodynamic efficiency results which showed that γ-CD-MgPc is more phototoxic than pure MgPc. It is also worth mentioning that after incubation for 4 h, cell structure was not affected, and no nuclear localization was observed at this incubation time.

## 4. Conclusions

The current research work demonstrated the successful preparation of inclusion complexes of natural (β-CD, γ-CD) and chemically modified cyclodextrins (HP-β-CD, Me-β-CD) with magnesium phthalocyanine (MgPc) via the kneading method. MgPc was successfully encapsulated with satisfactory inclusion efficiency values between 59% and 81%. The average size of the ICs ranged from 564 to 748 nm while the zeta potential ranged from −15 to −30 mV, indicating mild to high stability for the formed ICs. Moreover, the ICs were structurally characterized via FT-IR and NMR spectroscopy, which demonstrated the successful formation of the ICs, as well as that it is most likely that only part of the phthalocyanine molecule is located inside the respective cyclodextrin’s cavity. The Job’s plot study indicated that the stoichiometry of the produced ICs is cyclodextrin:MgPc = 2:1. Release studies of MgPc from the ICs at 37 °C and a pH of 7.4 indicated a burst effect in the first 20 min in which approximately 70% of the encapsulated MgPc was released. The release data had better fit on the Korsmeyer-Peppas kinetics, while the release mechanism is described as anomalous transfer.

The ICs were tested for their ability to produce ROS, presenting satisfactory ROS production when irradiated with a laser at 661 nm. The photodynamic therapy studies against a squamous carcinoma A431 cell line indicated a potent photosensitizing activity of MgPc (33% cell viability after irradiation for 3 min with 18 mW/cm^2^), as well as of the γ-CD-MgPc IC which was the most promising photosensitizing nanosystem, presenting the best PDT activity (cell viability 26% after 3 min of irradiation). Finally, intracellular localization studies indicated the enhanced cellular uptake of MgPc when encapsulated in the γ-CD cavity. Overall, CD-MgPc ICs can be considered as promising nanosystems for the sustained release of MgPc and the effective PDT cancer treatment.

## Figures and Tables

**Table 1:** Inclusion efficiency and process yield for ICs containing MgPc and different CDs.

Samples	MgPc:CD Ratio (mol)	%Inclusion Efficiency (%EE)	%Process Yield (%PY)
β-CD	1:1	81	82
HP-β-CD	1:1	97	76
γ-CD	1:1	67	79
Me-β-CD	1:1	59	69

**Table 2:** Size, PDI, and *ζ*-potential of the ICs.

Samples	Mean Hydrodynamic Radius (nm)	Polydispersity Index	*ζ*-Potential (mV)
β-CD-MgPc	564.5 ± 52.6	0.522 ± 0.024	−29.8 ± 1.2
HP-β-CD-MgPc	688.4 ± 105.1	0.552 ± 0.053	−17.7 ± 0.5
Me-β-CD-MgPc	746.1 ± 37.1	0.534 ± 0.014	−23.0 ± 1.6
γ-CD-MgPc	748.7 ± 52.0	0.566 ± 0.043	−14.9 ± 4.0

**Table 3:** Characteristic absorption bands of the tested compounds and products.

	Characteristic Absorption Bands (cm^−1^)										
	O-H Stretching	C-H Stretching	C-H Antisymmetric Stretching of CH_2_	C=C-C Stretching (Aromatic)	C-C Stretching Vibration (Isoindole)	O-H Bending	C-C Stretching (Pyrrole)	C-N Stretching (Pyrrole)	C-O Stretching (Secondary Alcohols)	Mg-N Stretching Vibration	C-H Out of Plane Deformation
MgPc	-	-	-	1525	1483	-	1333	1057	-	888	728
β-CD	3376	2924	1643	-	-	1414	-	-	1028	-	-
β-CD-MgPc	3411	2920	1646	1521	1482	1417	-	-	1027	-	727
HP-β-CD	3413	2926	1640	-	-	1468	-	-	1038	-	-
HP-β-CD-MgPc	3411	2926	1646	1528	1482	-	-	-	1030	-	727
Me-β-CD	3419	2931	1640	-	-	1457	-	-	1043	-	-
Me-β-CD-MgPc	3414	2926	1651	1521	1482	-	-	-	1044	-	725
HP-β-CD-MgPc (solvent free kneading)	3413	2925	1639	1525	1481	1454	1333	1057	1032	889	731
HP-β-CD-MgPc (physical mixture)	3413	2927	1639	1522	1481	1469	1331	1057	1032	889	730

**Table 4:** Chemical shift changes (∆*δ*) of ^1^H NMR (DMSO-*d_6_*, 600 MHz) signals of β-CD and β-CD in the ICs with MgPc.

Proton	Chemical Shifts (*δ*_1_) of β-CD Protons (ppm)	Chemical Shifts (*δ*_2_) of β-CD Protons in the β-CD-MgPc ICs (ppm)	∆*δ* = *δ*_2_ − *δ*_1_ (ppm)
H-1	4.826	4.802	0.024
H-2	3.3	3.276	0.024
H-3	3.632	3.608	0.024
H-4	3.353	3.333	0.020
H-5	3.559	3.534	0.025
H-6	3.632	3.608	0.024

**Table 5:** Chemical shift changes (∆*δ*) of ^1^H NMR (DMSO-*d_6_*, 600 MHz) signals of Me-β-CD and Me-β-CD in the ICs with MgPc.

Proton	Chemical Shifts (*δ*_1_) of Me-β-CD Protons (ppm)	Chemical Shifts (*δ*_2_) of Me-β-CD Protons in the Me-β-CD-MgPc ICs (ppm)	∆*δ* = *δ*_2_ − *δ*_1_ (ppm)
H-1	5.062	5.052	0.01
H-2	3.23	3.221	0.009
H-3	3.478	3.47	0.008
H-4	3.351	3.298	0.053
H-5	3.478	3.47	0.008
H-6	3.478	3.47	0.008

**Table 6:** Chemical shift changes (∆*δ*) of ^1^H NMR (DMSO-*d_6_*, 600 MHz) signals of HP-β-CD and HP-β-CD in the ICs with MgPc.

Proton	Chemical Shifts (*δ*_1_) of HP-β-CD Protons (ppm)	Chemical Shifts (*δ*_2_ of HP-β-CD Protons in the HP-β-CD-MgPc ICs (ppm)	∆*δ* = *δ*_2_ − *δ*_1_ (ppm)
H-1	4.980	5.000	−0.020
H-2	3.353	3.297	−0.056
H-3	3.734	3.722	0.024
H-4	3.353	3.297	−0.056
H-5	3.597	3.589	0.008
H-6	3.597	3.589	0.008
H-7	3.353	3.297	−0.056
H-8	3.734	3.722	0.024
H-9	1.009	0.994	0.015

**Table 7:** Chemical shift changes (∆*δ*) of ^1^H NMR (DMSO-*d_6_*, 600 MHz) signals of HP-β-CD and HP-β-CD in the physical mixture with MgPc.

Proton	Chemical Shifts (*δ*_1_) of HP-β-CD Protons (ppm)	Chemical Shifts (*δ*_2_) of HP-β-CD Protons in the HP-β-CD and MgPc Physical Mixture (ppm)	∆*δ* = *δ*_2_ − *δ*_1_ (ppm)
H-1	4.980	4.992	−0.012
H-2	3.353	3.311	0.042
H-3	3.734	3.741	−0.07
H-4	3.353	3.311	0.042
H-5	3.597	3.599	−0.002
H-6	3.597	3.599	−0.002
H-7	3.353	3.311	0.042
H-8	3.734	3.741	−0.007
H-9	1.009	1.012	−0.003

**Table 8:** R^2^ for different kinetic models based on the data from the release profile analysis.

IC	Zero Order	First Order	Higuchi	Korsmeyer-Peppas
	R^2^	R^2^	R^2^	R^2^
Me-β-CD-MgPc	0.314	0.238	0.586	0.806
β-CD-MgPc	0.371	0.252	0.653	0.823
HP-β-CD-MgPc	0.408	0.264	0.693	0.837

**Table 9:** Diffusion exponent n based on the Korsmeyer-Peppas equations.

IC	Korsmeyer-Peppas		Diffusion Exponent
	R^2^	Equation	
Me-β-CD-MgPc	0.806	y = 0.825x + 0.345	0.825
β-CD-MgPc	0.823	y = 0.819x + 0.325	0.819
HP-β-CD-MgPc	0.837	y = 0.814x + 0.306	0.814

## Data Availability

All data generated or analyzed during this study are included in this published article.

## Supplementary Materials

The following supporting information can be downloaded at: https://www.mdpi.com/article/10.3390/bioengineering10020244/s1, Figure S1: Possible orientation of the MgPc molecule towards the CDs’ cavity according to the Job’s Plot studies; Figure S2: ^1^H NMR of the: HP-β-CD-MgPc IC and the physical mixture of HP-β-CD and MgPc.
