# Supplementary material for: Ιnclusion Complexes of Magnesium Phthalocyanine with Cyclodextrins as Potential Photosensitizing Agents
Source: Bioengineering (Basel). 2023 Feb 13;10(2):244. doi: 10.3390/bioengineering10020244 (PMC9951963; doi:10.3390/bioengineering10020244)
Supplement: Supplementary file 1 [file bioengineering-10-00244-s001.zip › bioengineering-2168280-supplementary.pdf]

Supplementary Materials: Inclusion complexes of magnesium phthalocyanine with cyclodextrins as potential photosensitizing agents

Eleni Kavetsou, Charalampos Tsoukalas-Koulas, Annita Katopodi, Alexandros Kalospyros, Eleni Alexandratou and Anastasia Detsi*

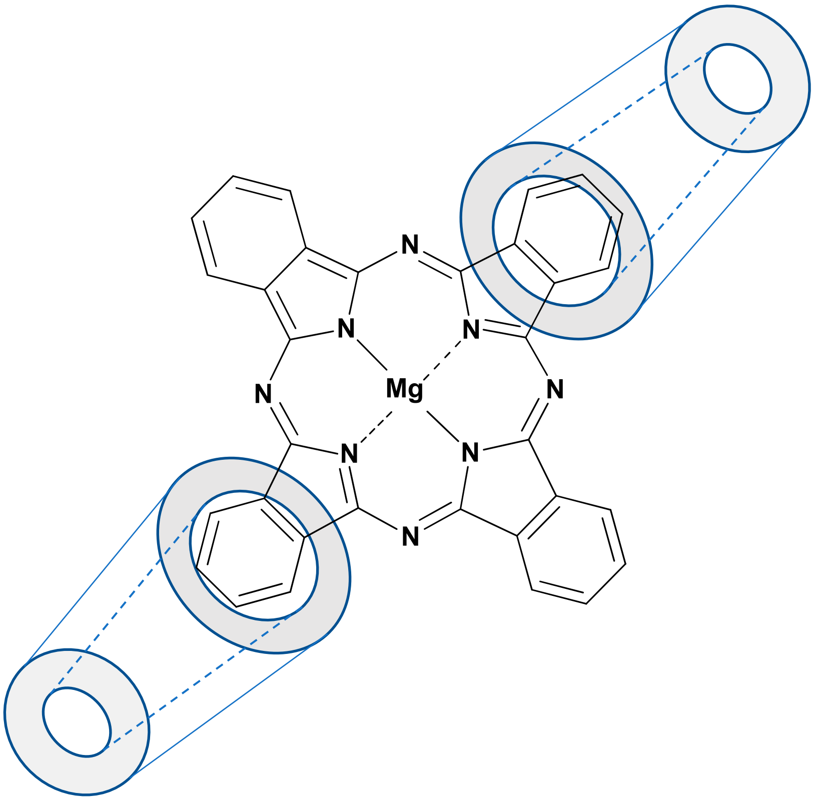

Figure S1. Possible orientation of the MgPc molecule towards the CDs' cavity according to the Job's Plot studies.

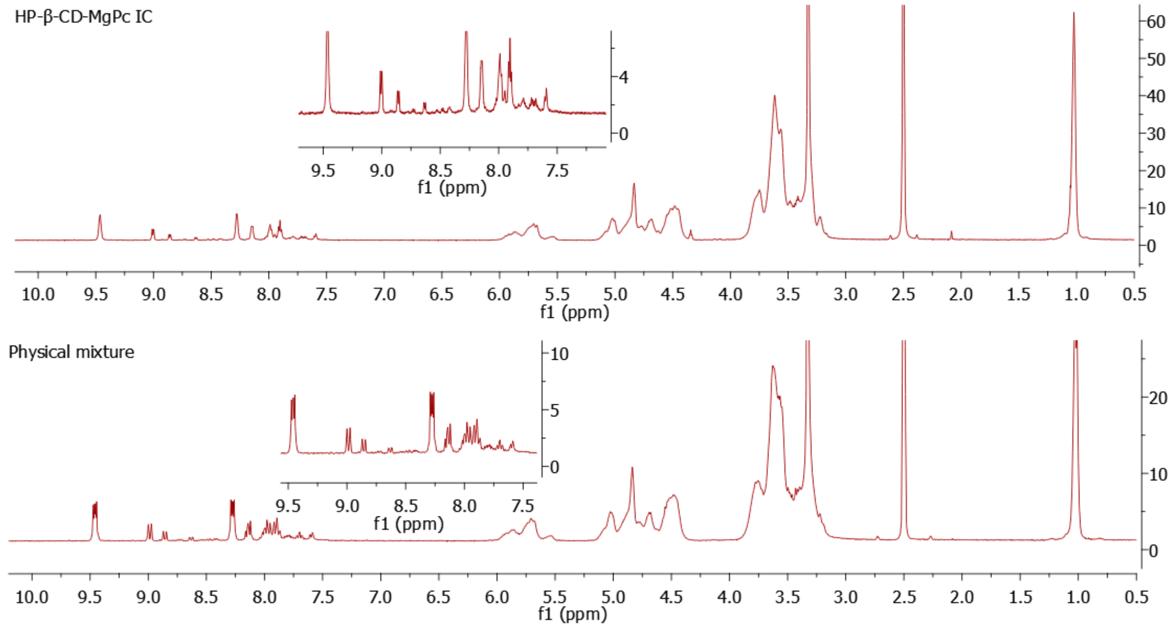

Figure S2. ^{1}H NMR of the: HP-β-CD-MgPc IC and the physical mixture of HP-β-CD and MgPc.
